# Prospective Study of Multiparametric Renal MRI for CKD Progression (AFiRM)

**DOI:** 10.1016/j.ekir.2026.106594

**Published:** 2026-05-16

**Authors:** Susan Francis, Charlotte E. Buchanan, Victoria Chester, Eleanor F. Cox, Martin Craig, Neeraj Dhaun, Alexander J. Daniel, Mark S. Gilthorpe, Keith A. Gillis, Matthew Hall, Parminder Judge, Philip A. Kalra, Andrew Lewington, Iosif A. Mendichovszky, David Morris, Andrew N. Priest, Neil S. Sheerin, Rachelle Sherman, Steven Sourbron, Maarten W. Taal, David L. Thomas, Nicholas M. Selby

**Affiliations:** 1 Sir Peter Mansfield Imaging Centre, University of Nottingham, Nottingham, UK; 2 Nottingham NIHR Biomedical Research Centre, University of Nottingham, Nottingham, UK; 3 Derby Clinical Trials Support Unit, University Hospitals of Derby and Burton NHS Foundation Trust (UHDB), Derby, UK; 4 Edinburgh Kidney Research Group, Centre for Cardiovascular Science, Queen’s Medical Research Institute, University of Edinburgh, Edinburgh, UK; 5 Obesity Institute, Leeds Beckett University, Leeds, UK; 6 Glasgow Renal and Transplant Unit, Queen Elizabeth University Hospital, Glasgow, UK; 7 Renal and Kidney Transplant Unit, Nottingham University Hospitals Trust, Nottingham, UK; 8 Clinical Trial Service Unit and Epidemiological Studies Unit, Nuffield Department of Population Health, University of Oxford, Oxford, UK; 9 Oxford Kidney Unit, Churchill Hospital, Oxford University Hospitals NHS Foundation Trust, Oxford, UK; 10 Renal Services, Salford Royal Hospital, Northern Care Alliance NHS Foundation Trust, Salford, UK; 11 Department of Renal Medicine, Leeds Teaching Hospitals NHS Foundation Trust, Leeds, UK; 12 Department of Radiology, Cambridge University Hospitals NHS Foundation Trust, Cambridge, UK; 13 Institute of Cellular Medicine, Faculty of Medical Sciences, Newcastle University, Newcastle, UK; 14 Department of Medical Imaging Physics, School of Medicine and Population Health, University of Sheffield, Sheffield, UK; 15 Centre for Kidney Research and Innovation, School of Medicine, University of Nottingham, Nottingham, UK; 16 Department of Renal Medicine, UHDB, Derby, UK; 17 Department of Translational Neuroscience and Stroke, UCL Queen Square Institute of Neurology, University College London, London, UK; 18 Department of Radiology, University of Cambridge, Cambridge, UK

**Keywords:** chronic kidney disease, CKD, multiparametric MRI, progression

## Abstract

**Introduction:**

Chronic kidney disease (CKD) has major population health implications, but current imaging approaches provide limited pathophysiological insights. We designed a study to evaluate renal multiparametric magnetic resonance imaging (MRI; mpMRI) as a tool to improve diagnosis, risk stratification, disease monitoring, and therapeutic decision-making in CKD.

**Methods:**

The application of functional renal MRI to improve assessment of CKD (AFiRM study; NCT04238299) is a multicenter, prospective, observational cohort study in people with CKD. Participants underwent renal mpMRI using the harmonized cross-vendor UK Renal Imaging Network: MRI Acquisition and Processing Standardization (UKRIN-MAPS) protocol at baseline and at 2-years. Clinical data are collected annually for 4 years with kidney failure outcomes assessed at 10 years. Primary objectives are to determine associations between MRI measures of kidney morphology, inflammation, fibrosis, perfusion, and oxygenation with CKD progression, and to quantify longitudinal changes in mpMRI measures. A biopsy substudy compares mpMRI measures with histology to explore mechanistic processes including fibrosis.

**Results:**

Across 9 centers, 420 participants (biopsy substudy *n* = 43) completed baseline assessments and mpMRI. Mean age is 55 years (SD 13), and 63.8% are male. Median estimated glomerular filtration rate (eGFR) is 39 ml/min per 1.73 m^2^ (IQR 29–53), median urine albumin-creatinine ratio (uACR) 47 mg/mmol (IQR 8.3–127.1), and median 5-year Kidney Failure Risk Equation score is 5.6% (IQR 1.0–20.8%). Most common CKD etiologies are IgA nephropathy (22.4%), CKD of unknown etiology (19.5%), and diabetic kidney disease (14.3%). Biopsy substudy participants were demographically similar to the full cohort.

**Conclusion:**

The AFiRM study has established a representative CKD cohort undergoing advanced renal mpMRI with long-term follow-up. This is an important resource to study how renal MRI measures and their longitudinal changes relate to CKD severity and progression.

## Graphical abstract

**Figure:**
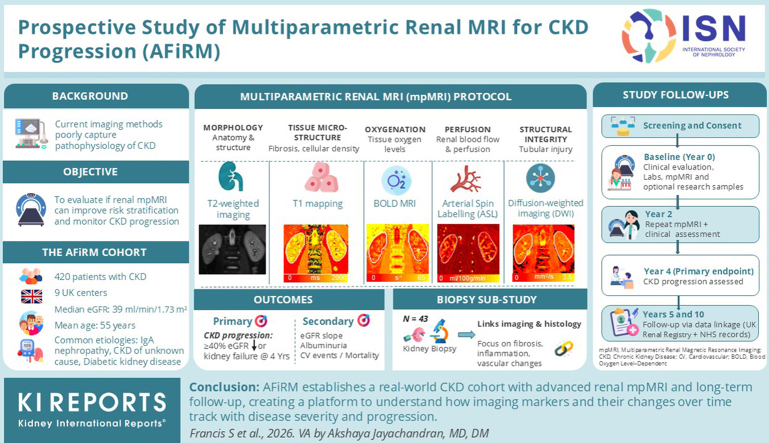

CKD affects 800 million people globally and has major implications for population health.^1^^,^^2^ The therapeutic landscape for CKD is rapidly changing, with new, effective therapies that target common mechanisms of progressive kidney damage as well as those that target disease-specific pathways.^3^ However, the trajectory of CKD progression varies significantly between individuals and disease etiologies, as does the magnitude of therapeutic response to different medication regimes.^4^ Determining the optimal combination of therapies is therefore becoming increasingly complex.^5^ At present, clinical decisions are heavily influenced by nonspecific markers of kidney function and glomerular damage (eGFR and albuminuria, respectively). Although these markers serve as descriptors of CKD severity, they only partially support prediction of future deterioration and do not distinguish pathophysiological processes of the primary kidney disease from the generic changes arising from nephron loss.^6^ Current clinical imaging techniques for the assessment and monitoring of CKD are limited and do not address these emerging challenges.

MRI has great potential to improve understanding and characterization of the mechanistic processes implicated in CKD progression.^7^^,^^8^ Structural and functional renal MRI measurements provide whole kidney assessments sensitive to changes in kidney morphology (T_1_-/T_2_-weighted and Dixon images), tissue microstructure (MR-relaxometry [T_1_/T_2_ relaxation time], diffusion weighted imaging [DWI]), oxygenation (blood oxygenation-level-dependent MRI), perfusion (arterial spin labelling [ASL]), and blood flow (phase contrast MRI). These measures can be collected in a single scan session, termed mpMRI. By avoiding ionizing radiation and gadolinium contrast, mpMRI allows serial assessments of the kidney, which is an advantage as compared with kidney biopsy that is rarely repeated; in some scenarios mpMRI can even reduce the need for biopsy.

However, knowledge gaps need to be addressed before renal mpMRI is ready for clinical adoption.^9^ To date, most research studies employing renal mpMRI have been cross-sectional, reporting associations of mpMRI measures with kidney function or histological measures of fibrosis.^10^^,^^11^ These results are useful to identify candidate MRI measures for prognostic modelling but do not inform how MRI measures are causally related to clinical outcomes, such as progression to kidney failure. Furthermore, the value of combining mpMRI measures in prognostic models is yet to be fully explored. This reflects the relatively small number of longitudinal studies that evaluated MRI associations for prognostic utility. Examples include that of Pruijm *et al.*^12^ who reported kidney oxygenation assessed using layer-based analysis of blood oxygenation level dependent MRI was independently associated with subsequent CKD progression in 112 participants. In contrast, a secondary analysis of a negative clinical trial did not find associations between blood oxygenation level dependent MRI or DWI (the apparent diffusion coefficient metric) and change in eGFR in a subgroup of 87 participants after adjusting for albuminuria.^13^ In a mixed cohort of transplant (*n* = 154) and patients with CKD (*n* = 43), Berchtold *et al.*^14^ reported the corticomedullary difference in apparent diffusion coefficient metric and cortical T_1_ were associated with an eGFR decline > 30% or dialysis initiation over 2.2 years median follow up, although the 2 measures were not additive.^15^ Using a similar definition of CKD progression, Shi *et al.*^16^ also reported an association with T_1_ in 119 patients with CKD stages G1 to 4. However, these studies are not conclusive as they employ only a subset of mpMRI measures, have modest sample sizes and only one incorporated serial mpMRI to assess the relationships between the change in MRI measures and CKD progression.^13^ As such, there is a clear need to evaluate comprehensive mpMRI in longitudinal studies with adequate sample size, length of follow-up, and robust end points of CKD progression.

To address this, we designed the AFiRM study to determine if renal mpMRI can provide structural and functional assessments of the kidneys that will provide prognostic information and ultimately guide treatment decisions. Here, we describe the study design, specific objectives, methods, and baseline participant characteristics.

## Methods

### Study Design

**Figure 1:**
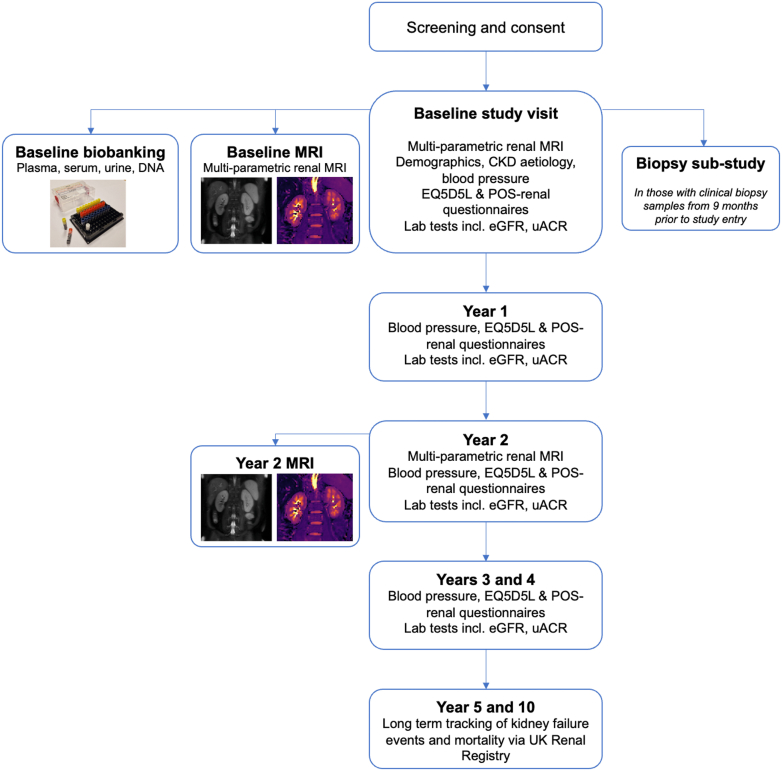
Summary of AFiRM study design. Participants were assessed with a multiparametric renal MRI protocol at baseline and year 2. Clinical data are collected at baseline and at annual follow-up visits for 4 years, after which kidney failure and survival will be determined at 5 and 10 years via data linkage with the UK Renal Registry. A biopsy substudy is performed on participants who have undergone recent kidney biopsy for clinical indications compares mpMRI measures with histology. AFiRM, application of functional renal MRI to improve assessment of CKD; mpMRI, multiparametric magnetic resonance imaging.

AFiRM is a multicenter, UK-based, prospective, observational cohort study in people with CKD of varying etiologies and severity. Participants were assessed with a comprehensive renal mpMRI protocol at 2 timepoints, baseline and after 2-years. Clinical data are collected at baseline and at annual follow-up visits for 4 years, after which kidney failure and survival will be determined at 5 and 10 years via data linkage with the UK Renal Registry. A mechanistic substudy in participants who have undergone recent kidney biopsy for clinical indications compares mpMRI measures with histology. The study was approved by the Health and Social Care Research Ethics Committee A and the UK Health Research Authority (IRAS ID: 271043), and all participants provided written consent before the start of any study procedures. The study design is summarized in Figure 1 and was registered at ClinicalTrials.gov before recruitment commenced (ID: NCT04238299).

### Study Setting

**Figure 2:**
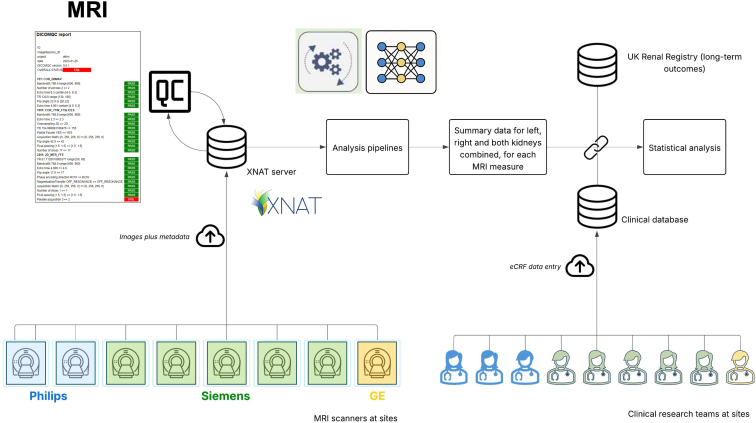
Data flow within the AFiRM study. Clinical and demographic data are uploaded by the clinical research teams to the clinical database (Dacima Software Inc). MR images and metadata are transferred to an online XNAT image repository (hosted by Dementias Platform UK) with quality control performed on upload using DICOM-QC. MRI data are then transferred to a high-performance computer for MR analysis to generate a database of MRI metrics which is then combined with the clinical database for statistical analysis. AFiRM, application of functional renal MRI to improve assessment of CKD; DICOM, digital imaging and communications in medicine-quality control; XNAT, extensible neuroimaging archive toolkit

**Figure 3:**
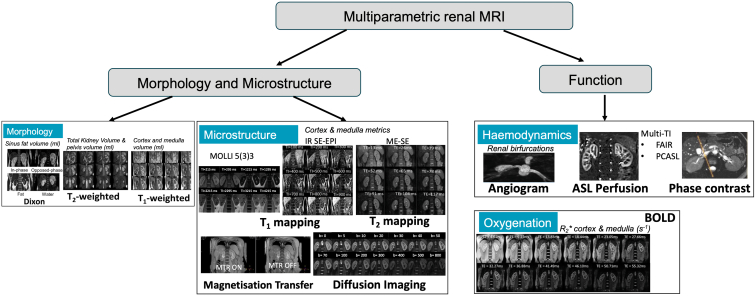
The “UKRIN-MAPS” renal mpMRI protocol used to assess kidney morphology and microstructure and function (hemodynamic and oxygenation) measures in of a nominal 1-hour scan duration. UKRIN-MAPS, UK Renal Imaging Network: MRI Acquisition and Processing Standardization

Participants were recruited from 9 UK secondary care nephrology centers. Preceding the AFiRM study, harmonization and standardization of 3 Tesla (3T) renal mpMRI measures across the 3 major MR vendor systems in the UK (GE, Philips, and Siemens) was achieved by the Medical Research Council-funded UKRIN-MAPS project. Figure 3 illustrates the “UKRIN-MAPS” renal mpMRI protocol which aligns with international technical recommendations for renal mpMRI.^17^, ^18^, ^19^, ^20^, ^21^, ^22^ This protocol was validated through a cross-vendor comparison in the same healthy individuals and a within-vendor repeatability study, described in detail elsewhere.^23^ This provided a validated protocol for the AFiRM multicenter study which uses 3T scanners from 5 Siemens sites, 1 GE site, and 2 Philips sites (1 Philips site scans participants from 2 nephrology centers), as shown in Figure 2.

### Outcomes

The objectives of AFiRM are as follows: (i) to establish associations between renal mpMRI measures (alone and in combination) and clinically meaningful outcomes; (ii) to describe the rate and magnitude of change in renal mpMRI measures over time, to aid understanding of how these could be used clinically to assess changes in underlying pathophysiology; and (iii) to gain evidence to show how MRI can detect and quantify mechanistic processes relevant to kidney diseases by relating measures to biopsy, e.g., fibrosis.

The primary clinical outcome is CKD progression at 4 years, defined as a ≥ 40% decline in eGFR from baseline or development of kidney failure (defined as persistent eGFR < 15ml/min per 1.73 m^2^, initiation of chronic dialysis, or receipt of kidney transplant). A 40% decline in eGFR was chosen as the more stringent definition that the US Food and Drug Administration and European Medicines Agency accept as a surrogate end point for kidney failure in clinical trials of kidney disease progression.^24^ Additional outcomes include the following: individual components of the primary outcome at each timepoint; eGFR trajectory (ml/min per 1.73 m^2^/yr); albuminuria; cardiovascular events; acute kidney injury events; and all-cause mortality.

### Eligibility Criteria

Eligibility criteria are detailed in Table 1.^25^ Participants have CKD with an eGFR of 15 to 59 ml/min per 1.73 m^2^, or if eGFR ≥ 60 ml/min per 1.73 m^2^ then with persistent albuminuria (uACR > 30 mg/mmol or urine protein-to-creatinine ratio > 50 mg/mmol). Because of the planned length of follow-up, the upper age limit was 75 years.

**Table 1:** Eligibility criteria for the AFiRM study

Inclusion criteria	Exclusion criteria
- Age 18–75 yrs - CKD category G3-4 (eGFR 15–59 ml/min per 1.73 m^2^) or CKD category G1-2 (eGFR ≥ 60 ml/min per 1.73 m^2^) with overt albuminuria (urine ACR > 30 mg/mmol or PCR > 50 mg/mmol) - Capable of giving informed consent	- Autosomal dominant polycystic kidney disease - Glomerulonephritis actively receiving immunosuppression or having done so within the preceding 90 d. - Multiple myeloma - Acute kidney injury (AKI) within the 90 d before consent (AKI defined as per KDIGO criteria^25^) - Solid organ transplant - Known single kidney - More than 5 simple cysts in 1 kidney on previous renal imaging - Contraindications to MRI - Current participation in other research studies that would conflict with this research study in the opinion of the principal investigator.

Exclusion criteria included known structural kidney abnormalities including autosomal dominant polycystic kidney disease, kidney transplant, single kidney, or > 5 cysts in 1 kidney. Scenarios in which acute changes in kidney function were anticipated were also excluded (acute kidney injury within preceding 90-days, myeloma, or glomerulonephritis receiving immunosuppression), as were those with contraindications to MRI (e.g., metal implants, fragments or devices, claustrophobia).

### Data Collection

#### Clinical Data

A range of demographic data, medical history including etiology of CKD, other comorbidities, and medications were collected at baseline, as well as blood pressure measurement, quality of life (EuroQol 5-Dimension 5-Level questionnaire) and symptom (Palliative Care Outcome Scale-Renal) questionnaires. Blood and urine tests for routine biochemistry and hematology were taken and measured in local NHS laboratories (including uACR and eGFR-creatinine, calculated using the CKD-EPI 2009 equation without correction for ethnicity in line with current UK guidance^26^). Any changes in medical status and medications were captured at annual clinical visits, alongside repeat blood and urine testing, quality of life and symptom questionnaires, and blood pressure measurement.

#### Renal mpMRI Protocol

Renal mpMRI was coordinated by the central MRI site at the Sir Peter Mansfield Imaging Centre that is responsible for MRI acquisition including training the MRI site teams on the protocol, quality control, and analysis. Before commencing the study, MRI sites performed scans on the International Society for Magnetic Resonance in Medicine and the National Institute of Standards and Technology’s MR phantom (CaliberMRI, Boulder, CO)^27^ and a healthy volunteer to ensure image quality. Renal mpMRI is performed at baseline and 2 years, with scan sessions scheduled within 6 weeks of the corresponding clinical visit. Participants are requested to refrain from eating or drinking for 2 hours preceding their MRI scan.

The “UKRIN-MAPS” renal mpMRI protocol assesses kidney morphology, microstructure, oxygenation, blood flow, and perfusion in a nominal 1-hour duration, as shown in Figure 3 and summarized below:

Localizer scans: Used to plan slice placement with all subsequent renal mpMRI measures planned with matched coronal-oblique orientation to allow spatial combination of data.

Structural scans comprising T_1_-/T_2_-weighted^28^ and Dixon: T_2_-weighted scans for segmentation of whole kidney, kidney parenchyma, pelvis, and cysts; T_1_-weighted scans to segment cortex and medulla; Dixon scan to assess kidney sinus fat.

B_0_ and B_1_ maps: To assess magnetic field homogeneity in the kidney, used to correct mapping data.

BOLD R_2_∗ mapping: Acquired using a multiecho fast field echo scheme from which to fit R_2_∗ as a measure of cortical and medullary oxygenation.

T_1_ relaxation time mapping to assess microstructure: Collected using a shortened modified look-locker inversion recovery^29^ and respiratory-triggered inversion recovery spin-echo echo-planar imaging^30^ scheme.

T_2_ relaxation time mapping to assess microstructure: Collected using a multiecho spin echo scheme.^31^

DWI to assess microstructure: Respiratory-triggered at 13 b-values in 6 directions.

Magnetization transfer ratio to assess microstructure: Collected with a single MT pulse OFF and ON to selectively saturate macromolecules.

Angiogram and phase contrast-MRI: A noncontrast-enhanced angiogram is used to determine renal artery bifurcations and plan phase contrast-MRI slice placement. Renal artery blood flow in the right and left renal arteries is measured using phase contrast-MRI and combined with kidney volume to compute global perfusion.

ASL for perfusion: Collected using either a flow-alternating-inversion-recovery or pseudo-continuous ASL scheme,^22^ both with spin-echo echo-planar imaging readout.

A standardized scan naming convention is used across all MRI sites. All sites follow their local incidental findings policy informing participants and their medical teams of findings of clinical significance. Four sites underwent MR scanner software upgrades between the baseline and 2-year MRI scan; 1 site underwent an upgrade during baseline data collection and so repeat phantom and healthy volunteer scan quality control procedures were collected after the upgrade.

#### MRI Data Management and Analysis

Images were transferred to an online extensible neuroimaging archive toolkit image repository (Fig.2) with quality control performed on upload using digital imaging and communications in medicine-quality control, an open-source extensible neuroimaging archive toolkit comparing digital imaging and communications in medicine image tags to predefined values. Data were then transferred to a high-performance computer.

Full details of the MRI analysis pipeline are provided elsewhere.^32^^,^^33^ This comprises local machine-learning U-Net segmentation networks to generate masks of renal parenchyma and renal cysts from the T_2_-weighted scan,^34^ and whole kidney segmentation (parenchyma, pelvis, and cysts).^35^ Parenchyma and whole kidney masks are interrogated for volume and shape radiomics measures using Pyradiomics.^36^ U-nets are applied to shortened modified look-locker inversion recovery T_1_-maps to define the cortex and medulla.^37^ All masks are reviewed and manually corrected if required using MRIcroGL (https://www.nitrc.org/projects/mricrogl). R_2_∗ maps are generated using an exponential model, T_2_-maps are generated using a StimFit model to account for B_1_-inhomogeneity.^31^ Distortion correction is applied to the spin-echo echo-planar imaging-based data (DWI, ASL, T_1_ spin-echo echo-planar imaging) and model-driven-registration used to realign T_1_-mapping, DWI, magnetization transfer ratio, and ASL data. T_1_ mapping is performed accounting for B_1_-inhomogeneity, DWI data are used to compute ADC, fit to an intravoxel incoherent motion model (D, D∗, f) and estimate fractional anisotropy. The intravoxel incoherent motion model uses a segmented 2-step approach: first estimating D at high b-values via weighted linear least squares iteratively on log-transformed signal, then fitting D∗ and f across all b-values with D fixed. Magnetization transfer ratio maps are formed from subtraction of MT ON and MT OFF datasets, and ASL data are fit to a perfusion model. Cortex and medulla masks are applied to each mpMRI quantitative map to compute metrics (median/90th percentile and SD/full-width-half-maximum to assess heterogeneity) within the left and right kidney and combined across kidneys, and to estimate the corticomedullary difference.

#### Biosamples

At baseline, aliquots of plasma, serum, DNA, and urine were collected for long-term storage. Blood tubes were kept at room temperature for 45 to 120 mins before being centrifuged at 2000G for 10 mins at 20 ^°^C. 10 ml urine samples were centrifuged at 2000G for 10 mins at 20 ^°^C as soon as possible. After aliquoting into 0.2 ml bar-coded tubes, samples were placed into a -80 ^0^C freezer before transfer to Leeds Biobanking and Sample Processing Laboratory. Samples will allow ancillary studies of the relationship between renal mpMRI measures and biomarkers of specific progression mechanisms (e.g., fibrosis).

#### Biopsy substudy

We estimated ∼10% of recruited participants would have undergone routine clinical care kidney biopsy in the 9-months before study entry. Nine months was chosen as a pragmatic time window to allow sufficient recruitment to the substudy balanced against time between biopsy and baseline MRI. After consent, kidney biopsy tissue stained in clinical laboratories and residual kidney biopsy tissue embedded in paraffin blocks were transported to a central laboratory (the Human Biomaterials Resource Centre, University of Birmingham). Digital images were taken from sections with standard stains and undergo quantitative analysis, and additional sections cut for immunohistochemical staining (CD34 positive endothelial cells, Collagen III, and CD45 leukocyte common antigen positive cells). This substudy aims to assess whether MRI measures (alone or in combination) can discriminate different pathological processes related to CKD progression including fibrosis, inflammation, and vascular density.

### Sample Size

Previous studies show event rates of CKD progression in similar secondary care cohorts range from 13% kidney failure at 26 months,^38^ 25% kidney failure or eGFR decline of > 50% at 5.7 years,^39^ to 34.7% kidney failure at 4.7 years.^40^ Recruitment targets were based on a sample size of 450, which with a conservative estimate of 10% event rate of CKD progression, would generate confidence intervals of ±2.8% around simple cross-sectional estimates.

### Statistical Analysis

Descriptive statistics were used to outline baseline clinical characteristics of the study cohort. Continuous normally distributed data were presented as mean (SD) else median (inter-quartile range[IQR]). Categorical data were presented as counts and percentages.

Analyses will be conducted at key stages. At baseline, associations between each mpMRI measure and clinical variables of CKD severity (eGFR, uACR) will be assessed, studying potential confounding variables e.g. age, sex, ethnicity, smoking status, previous eGFR, CKD etiology, comorbidity, and medications. At year 2, the change in mpMRI measures will be assessed. At years 2 and 4, prognostic models will be built using baseline clinical and mpMRI measures to predict subsequent kidney disease progression and other clinical outcomes. Prognostic models with only baseline clinical data will be contrasted to models that incorporate mpMRI data.

All clinical and mpMRI predictors identified as having utility to discriminate severity or progression of kidney disease, or both, will be assessed and quantified for their causal impacts. This will be informed by a directed acyclic graph that encodes *a priori* known or hypothesized data generating mechanisms and causal relationships with downstream outcomes. Causal analyses will investigate what causes patients to be well differentiated by clinical and/or mpMRI measures to assess patient trajectories over the study duration. Multilevel and latent growth curve models will be used to accommodate both within and between patient heterogeneity in both absolute levels and changes in kidney disease. Latent subgroup analysis will examine if patient trajectories for the various clinical and mpMRI measures determine subgroups of patients. We will explore how longitudinal changes in the clinical and mpMRI measures relate to each other (i.e., how eGFR and uACR change over time and which more consistently track to MRI measures). Missing data methods will ensure that careful assessment and compatibility with analytical models is undertaken.^41^

### Patient and Public Involvement and Engagement

Three public involvement and engagement (PPIE) representatives from the lead investigator’s center and Kidney Research UK had an active role in grant writing, as well as contributing to preparation of the study protocol and participant facing documents. At study outset, an AFiRM PPIE group was formed including those previously involved alongside new members with experience of participating in MRI research studies. This group helped plan AFiRM study visits, for example aspects of participant transport and ways to improve convenience of attending clinical visits. PPIE representatives contributed to study oversight as part of the study steering committees. The clinical trials support unit produces quarterly participant newsletters that are disseminated by local study teams to inform of study progress. We have held a webinar for study participants, which included a presentation by a PPIE member on their involvement.

### Recruitment and Cohort Description

Recruitment commenced in June 2021, with a stop-go pilot of site set-up and enrolment of 50 participants successfully completed in February 2022. Recruitment to the study was completed in November 2023 and year-2 MRI scanning in March 2026. Year-4 study visits are due to be completed in November 2027.

**Figure 4:**
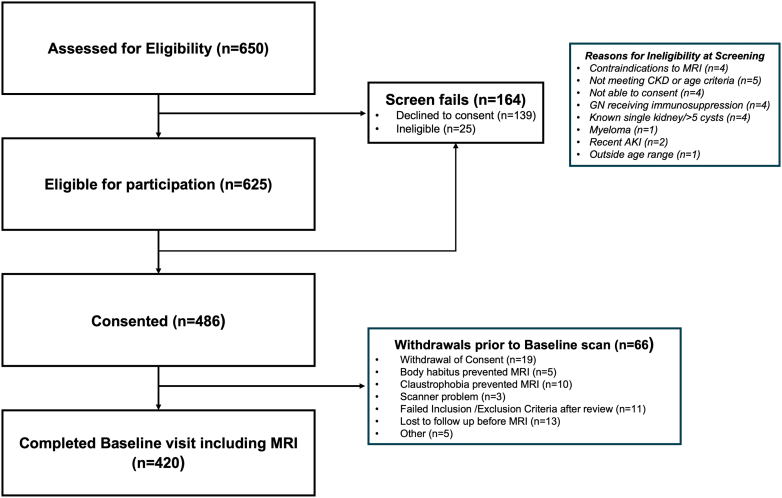
Consort diagram for the AFiRM study showing participant flow through screening and consent resulting in the 420 participants completing the Baseline study assessment using MRI. AFiRM, application of functional renal MRI to improve assessment of CKD; MRI, magnetic resonance imaging.

**Figure 5:**
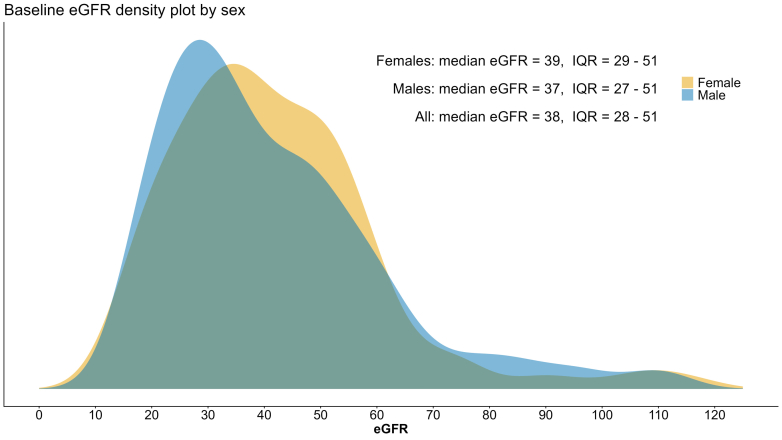
Distribution of baseline eGFR values across the AFiRM cohort disaggregated by sex (eGFR values shown in ml/min per 1.73 m^2^). AFiRM, application of functional renal MRI to improve assessment of CKD; eGFR, estimated glomerular filtration rate.

**Figure 6:**
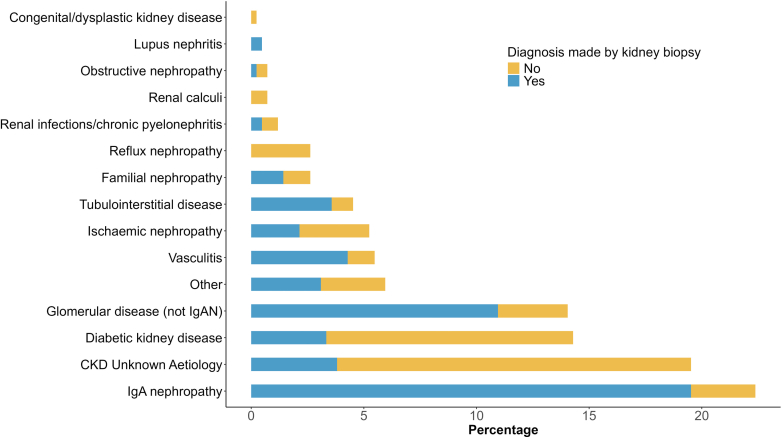
Prevalence of primary renal diseases in the AFiRM cohort, showing those whose diagnosis was made by kidney biopsy. AFiRM, application of functional renal MRI to improve assessment of CKD.

A total of 650 people with CKD were screened of whom 486 met eligibility criteria and consented to participate. Of these, 420 completed the baseline study visit including the first MRI scan and form the main study cohort. Participant flow and reasons for nonparticipation are shown in Figure 4. A description of the 420 participants’ baseline characteristics is shown in Table 2. Mean age is 55 years (SD 13), 268 (63.8%) are male, and 87.1% are of white ethnicity. Median eGFR is 39 ml/min per 1.73m^2^ (IQR 29–53) and median uACR is 47 mg/mmol (IQR 8.3–127.1). Distribution of participants across the Kidney Disease: Improving Global Outcomes classification is shown in Table 3, with most participants having eGFR values < 60 ml/min per 1.73 m^2^ (87%) and uACR values > 3.0 mg/mmol (86.4%). The distribution of eGFR values is similar between males and females (Figure 5). The median Kidney Failure Risk Equation score is 5.6% in 5 years, with wide IQR (1.0–20.8%). The proportion of participants across etiologies of CKD is shown in Figure 6. The most common primary renal disease is IgA nephropathy in 94 (22.4%), followed by CKD of unknown etiology in 82 (19.5%), then diabetic kidney disease in 60 (14.3%). Across the entire cohort, 224 (53.3%) have primary renal disease established by kidney biopsy. In terms of comorbidity, 263 (62.6%) have hypertension, and 79 (18.8%) have diabetes that was not the primary cause of CKD. At baseline, renin-angiotensin-aldosterone system inhibitors were taken by 316 (75.2%) participants, and sodium-glucose cotransporter-2 inhibitors by 175 (41.6%).

**Table 2:** AFiRM study participant characteristics at baseline

Participant characteristics	Number (%)	Mean (SD) or median (IQR)	Data completeness (number available, %)
Age (yrs)		55 (13)	420 (100%)
Sex			
Male:Female	268 (64%): 152 (36%)		
Ethnicity			
White	366 (87.1%)		
South Asian	23 (5.5%)		
Black	13 (3.1%)		
Chinese	2 (0.5%)		
Mixed	6 (1.4%)		
Other	10 (2.4%)		
Blood pressure (mmHg)			417 (99.3%)
Systolic		137.8 (21.3)	417 (99.3%)
Diastolic		81.5 (12.1)	
CKD etiology			
IgA nephropathy	94 (22.4%)		
CKD of unknown etiology (CKDx)	82 (19.5%)		
Diabetic kidney disease	60 (14.3%)		
Glomerular disease (other than IgAN)	59 (14.0%)		
Vasculitis	23 (5.5%)		
Ischemic nephropathy	22 (5.2%)		
Tubulointerstitial disease	19 (4.5%)		
Familial nephropathy	11 (2.6%)		
Reflux nephropathy	11 (2.6%)		
Renal infections/chronic pyelonephritis	5 (1.2%)		
Obstructive nephropathy	3 (0.7%)		
Renal calculi	3 (0.7%)		
Lupus nephritis	2 (0.5%)		
Congenital/dysplastic kidney disease	1 (0.2%)		
Other	25 (6.0%)		
Comorbidity			
Hypertension	263 (62.6%)		
Diabetes (not PRD)	79 (18.8%)		
Cardiovascular disease	31 (7.4%)		
Cerebrovascular disease	8 (1.9%)		
COPD	7 (1.7%)		
Liver disease	6 (1.4%)		
Other	261 (62.1%)		
Smoking			
Current	32 (7.6%)		
Previous	150 (35.7%)		
Never	238 (56.7%)		
eGFR (ml/min per 1.73 m^2^)			417 (99.3%)
12 mos before study		39 (29 – 53)	
At study entry		37 (28 – 51)	420 (100%)
uACR (mg/mmol)			242 (57.6%)
12 mos before study		39.6 (6.9 – 114.7)	399 (95%)
At study entry		47.0 (8.2 – 127.1)	
CRP (mg/dl)		2.0 (0.9 – 4.7)	412 (98.1%)
Hemoglobin (g/L)		132 (19.0)	417 (99.3%)
Albumin (g/L)		38 (5)	419 (99.8%)
HbA1c (mmol/mol)		43.6 (14.4)	398 (94.8%)
Calcium (mmol/l)		2.4 (0.1)	419 (99.8%)
Phosphate (mmol/l)		1.1 (0.2)	416 (99.0%)
Cholesterol (mmol/l)			410 (97.6%)
HDL		1.4 (0.5)	411 (97.9%)
LDL		4.8 (1.4)	
KFRE score (5-yr risk,%)			
Overall		5.6 (1.0 – 28.0)	399 (95.0%)
Male		6.2 (1.2 – 21.6)	257
Female		4.3 (0.8 – 17.9)	142

COPD, chronic obstructive pulmonary disease; eGFR, estimated glomerular filtration rate; HDL, high-density lipoprotein cholesterol; IQR, interquartile range; KFRE, Kidney Failure Risk Equation; LDL, low-density lipoprotein cholesterol; PRD, primary renal disease; uACR, urinary albumin to creatinine ratio.

For 224 (53.3%) participants, CKD etiology was established by kidney biopsy.

**Table 3:** Participant baseline distribution across KDIGO CKD stages

CKD stages		A1	A2	A3	Total
		(uACR < 3)	(uACR 3–30)	(uACR > 30)	
G1	(GFR > 90)	0 (0.0%)	1 (0.3%)	13 (3.3%)	14 (3.5%)
G2	(GFR 60–90)	2 (0.5%)	7 (1.8%)	29 (7.3%)	38 (9.5%)
G3a	(GFR 45–59)	33 (8.3%)	21 (5.3%)	48 (12.0%)	102 (25.6%)
G3b	(GFR 30–44)	17 (4.3%)	41 (10.3%)	68 (17.0%)	126 (31.6%)
G4	(GFR 15–29)	6 (1.5%)	40 (10.0%)	71 (17.8%)	117 (29.3%)
G5	(GFR < 15)	0 (0.0%)	0 (0.0%)	2 (0.5%)	2 (0.5%)
Total		58 (14.5%)	110 (27.6%)	231 (57.9%)	399 (100.0%)

CKD, chronic kidney disease; GFR, estimated glomerular filtration rate (ml/min per 1.73 m^2^); KDIGO, Kidney Disease: Improving Global Outcomes; uACR, urine albumin to creatinine ratio (mg/mmol).

Of the 420 participants, 409 (97.3%) consented to collection of additional samples for long-term storage of whom 394 (96.3%) had a complete set of aliquots. Sample processing achieved 98% of blood samples and 93% of urine samples centrifuged within 2-hours of collection, and 99.8% of plasma/serum aliquots and 99.5% of urine aliquots frozen within 4-hours of collection. 43 participants form the biopsy substudy, with similar age (54 yrs, SD 14), sex (60.5% male), eGFR (38 ml/min per 1.73 m^2^, IQR 32–58), and uACR (64 mg/mmol, IQR 26–211) to the main cohort.

## Discussion

The AFiRM study aims to provide new insights into how advanced renal mpMRI can improve understanding and assessment of patients with CKD. It has a significantly larger sample size than previous MRI studies in CKD that will improve precision of estimates and enable subgroup analyses (e.g., CKD etiologies).^12^^,^^13^^,^^16^ Longitudinal follow-up for 10 years will increase the number of observed outcome events, in particular CKD progression, and allow detection of meaningful changes in eGFR over time. The baseline characteristics of participants show a balanced distribution across eGFR and uACR, as well as a range of CKD etiology categories, reflecting the broad eligibility criteria that were deliberately sought. This is also reflected in the wide distribution of kidney failure risk equation scores. Sex and ethnicity are similar to other UK CKD cohort studies (e.g., NURTuRE-CKD) and patients are recruited from a range of centers across the UK that together enhance generalizability of results.^42^

The multicenter design was enabled by adopting the “UKRIN-MAPS” protocol of MR sequences harmonized across the 3 MR vendor platforms. A previous healthy volunteer study demonstrated low variance in measures collected across MR vendor platforms, of key importance when assessing cross-sectional characteristics across 9 centers. Centralizing MRI data analysis allows quality control of measures, standardization of segmentations and mpMRI pipelines. This approach forms a ‘blueprint’ for future multicenter renal MRI research.

The mpMRI protocol collects a much broader range of quantifiable measures as compared with previous studies,^12^^,^^13^^,^^16^ including measures sensitive to changes related to kidney morphology, microstructure, hemodynamics, and oxygenation. The range of MRI measures will allow for comprehensive assessment of multiple pathophysiological processes relevant to CKD, offering new opportunities for patient assessment,^9^ and the biopsy substudy will allow direct comparisons between histological measures and mpMRI. Results will also allow the most important mpMRI measures to be determined, and identifying redundancy in MRI measures will allow for shorter, more efficient scan protocols better suited for clinical translation which can be further shortened using vendor-provided deep learning methods (such as AIR Recon DL, SmartSpeed, and Deep Resolve). Crucially, AFiRM includes a repeat mpMRI scan after 2 years, allowing assessment of how mpMRI measures change relative to CKD severity and progression. A further strength comes from the statistical analysis approach based on causal inference methods, which will move beyond identification of associations and allow for more rigorous estimation of causal and effect relationships by controlling for confounding, including the effect of CKD therapies, and provide more reliable inferences from the imaging and clinical data combined.

In summary, the AFiRM study will provide important novel insights into how renal mpMRI can be used to improve clinical assessment of CKD severity and its progression as well as exploration of underpinning causal mechanisms. Results will help renal MRI advance from its current position as a research tool towards clinical application.

## Disclosure

All the authors declared no competing interests.

## Supplementary Material

Supplementary file (PDF) STROBE Checklist.
